# Supplementary figures and images for: Pulsed-electromagnetic-field induced osteoblast differentiation requires activation of genes downstream of adenosine receptors A2A and A3
Source: PLoS One. 2021 Feb 25;16(2):e0247659. doi: 10.1371/journal.pone.0247659 (PMC7906300; doi:10.1371/journal.pone.0247659)

BSPI/SPP1/Osteopontin (66kDa)  
Panel 1

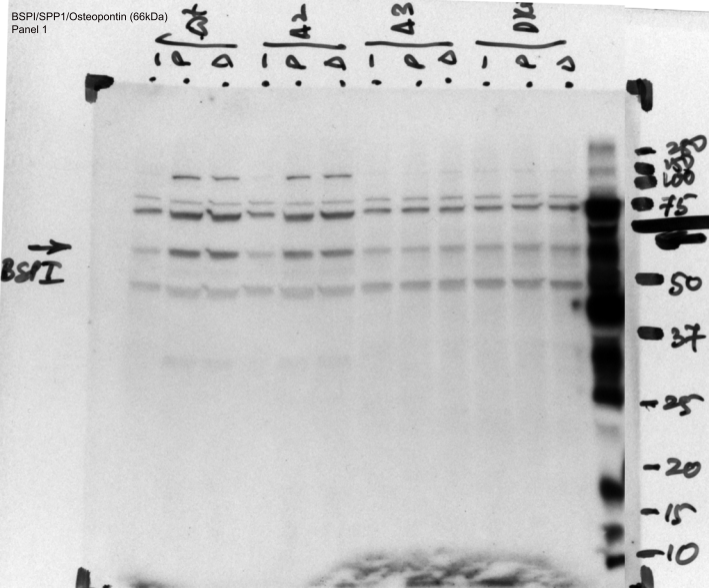

82 →

1 2 3 4 5 6 7 8 9

200  
100 150  
75  
50  
37  
25  
20  
15  
10

Osteocalcin (10kDa)  
Panel 3

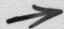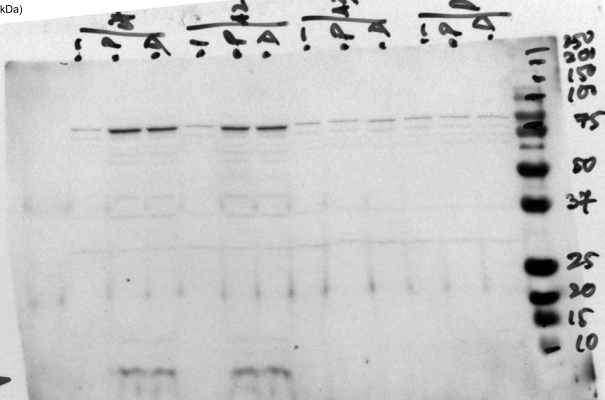

GAPDH (36KDa)  
Panel 4

gapdh  
→

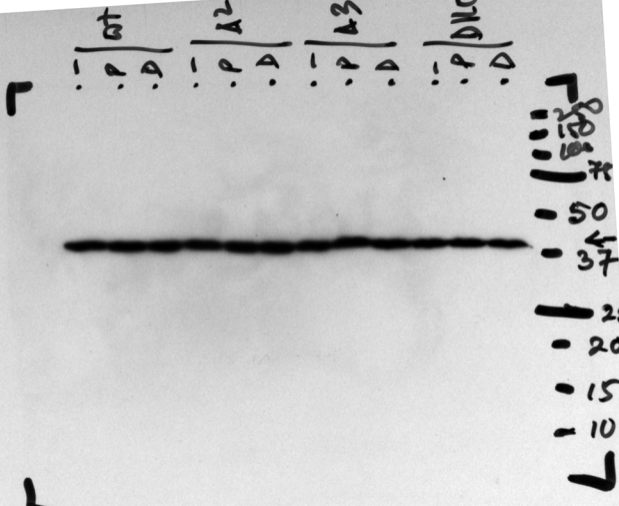

Supplement: S1 Raw images — (PDF) [file pone.0247659.s001.pdf]
