# Supplementary material for: Pulsed-electromagnetic-field induced osteoblast differentiation requires activation of genes downstream of adenosine receptors A2A and A3
Source: PLoS One. 2021 Feb 25;16(2):e0247659. doi: 10.1371/journal.pone.0247659 (PMC7906300; doi:10.1371/journal.pone.0247659)
Supplement: S1 File — (PDF) [file pone.0247659.s002.pdf]

Figure 1A

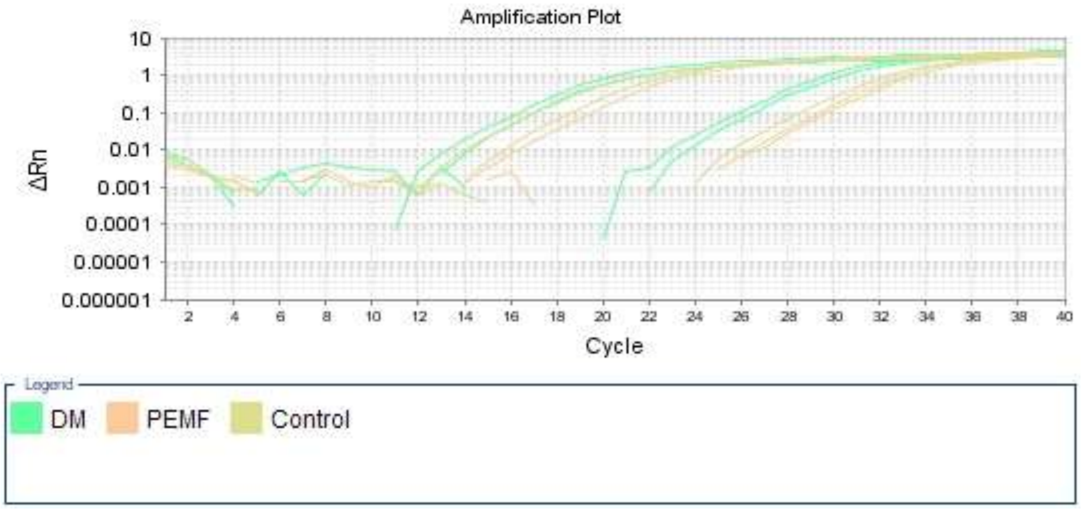

Figure 1B Alpl cFOS Pthr1 Runx Sp7 Ibsp

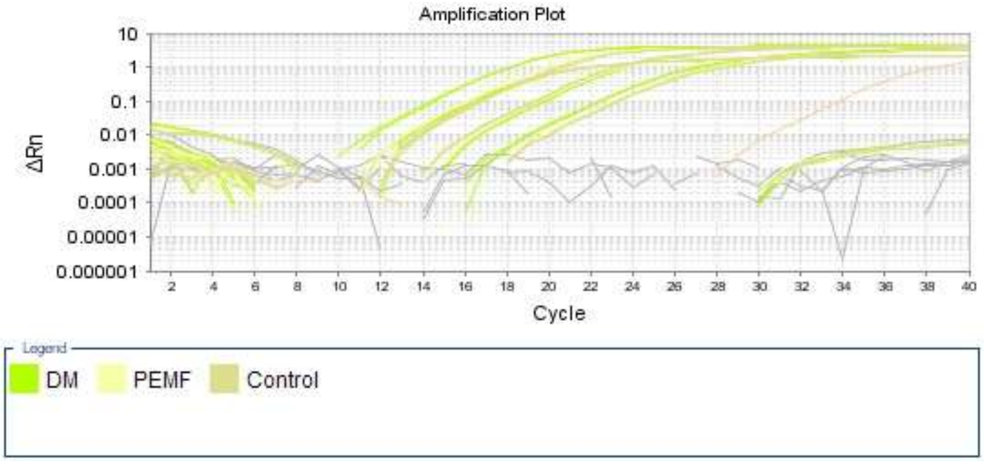

Figure 1B Osteocal BSPI

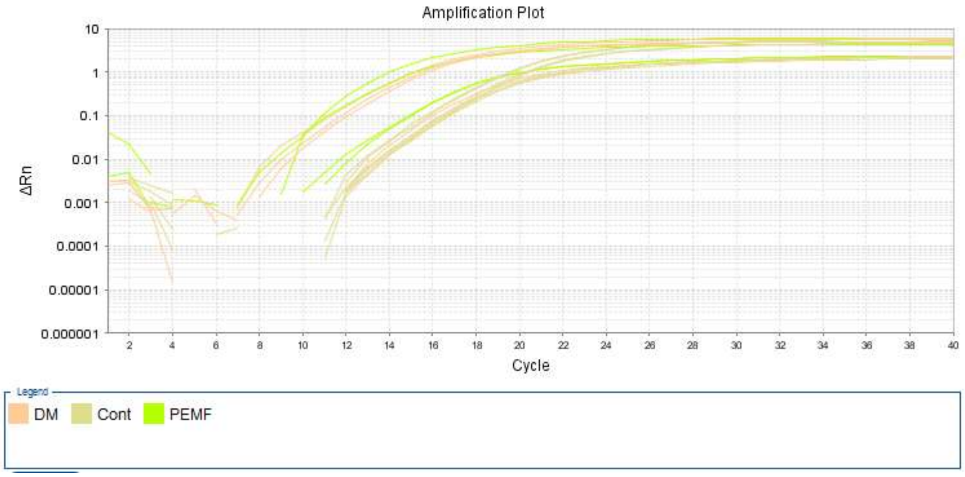

Figure 1 Data.

Block Type	96well
Chemistry	TAQMAN
Experiment File Name	H:\Cell Mol Med JoeD\Real-Time PCR\MC3T3 Alpl Pemp 3.eds
Experiment Run End Time	2020-06-14 18:59:50 PM EDT
Instrument Type	steponeplus
Passive Reference	ROX

Sample Name	Target Name	Reporter	Cт	ΔCт	ΔCт Mean	ΔΔCт	Fold	Mean Fold	SD
Control	Alpl	FAM	31.8	14.76	14.725	0.035	0.98	1.000294	0.034312
Control	Alpl	FAM	32.74	14.69		-0.035	1.02		
PEMF	Alpl	FAM	33.08	14.03	13.995	-0.695	1.62	1.659127	0.056912
PEMF	Alpl	FAM	33.32	13.96		-0.765	1.70		
DM	Alpl	FAM	30.5	13.4	13.465	-1.325	2.51	2.397389	0.152651
DM	Alpl	FAM	30.33	13.53		-1.195	2.29		
Control	gapdh	VIC	17.04						
Control	gapdh	VIC	18.05						
PEMF	gapdh	VIC	19.05						
PEMF	gapdh	VIC	19.36						
DM	gapdh	VIC	17.1						
DM	gapdh	VIC	16.8						

	mean	sd
Control	1	0.03
PEMF (4 hours/day)	1.66	0.06
DM	2.4	0.15

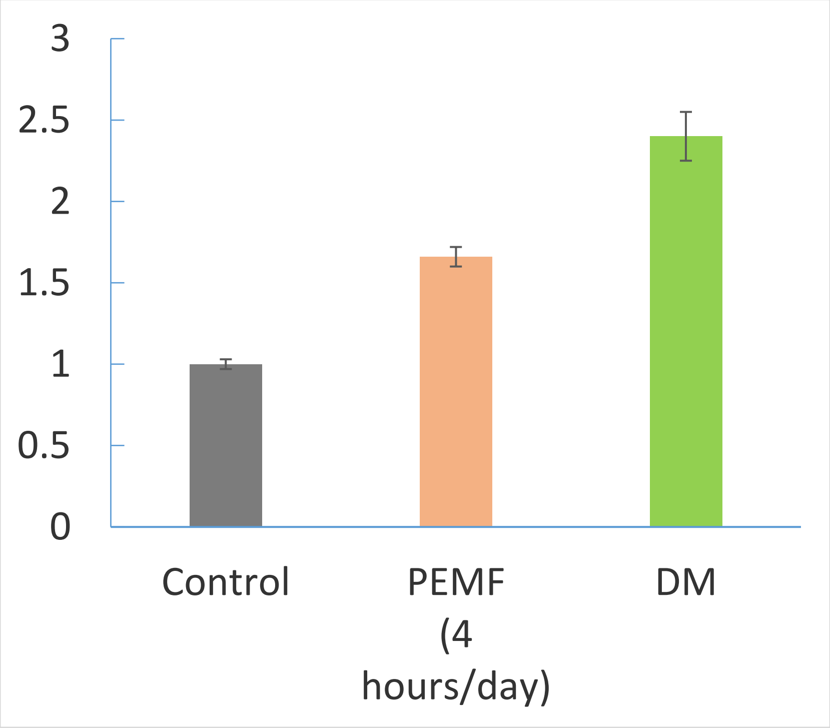

Block Type	96well

Chemistry	TAQMAN
Experiment File Name	H:\Cell Mol Med JoeD\Real-Time PCR\Extended.eds
Experiment Run End Time	2019-01-17 18:59:50 PM EDT
Instrument Type	steponeplus
Passive Reference	ROX

Sample Name	Target Name	Reporter	Cт	ΔCт	ΔCт Mean	ΔΔCт	Fold	Mean Fold	SD
Control	Alpl	FAM	24.8	6.86	6.815	0.045	0.97	1.000486	0.044119
Control	Alpl	FAM	24.74	6.77		-0.045	1.03		
PEMF	Alpl	FAM	23.08	4.84	4.985	-1.975	3.93	3.573343	0.506202
PEMF	Alpl	FAM	23.32	5.13		-1.685	3.22		
DM	Alpl	FAM	22.5	4.78	4.91	-2.035	4.10	3.760304	0.477896
DM	Alpl	FAM	22.73	5.04		-1.775	3.42		
Control	gapdh	VIC	17.94	17.955					
Control	gapdh	VIC	17.97	18.105					
PEMF	gapdh	VIC	18.24	18.215					
PEMF	gapdh	VIC	18.19	17.955					
DM	gapdh	VIC	17.72	17.705					
DM	gapdh	VIC	17.69	17.705					
Control	cFos	FAM	31.8	13.86	14.315	-0.455	1.37	1.050146	0.453448
Control	cFos	FAM	32.74	14.77		0.455	0.73		
PEMF	cFos	FAM	31.03	12.79	12.91	-1.525	2.88	2.657344	0.311867
PEMF	cFos	FAM	31.22	13.03		-1.285	2.44		
DM	cFos	FAM	30.98	13.26	13.1	-1.055	2.08	2.335699	0.364839
DM	cFos	FAM	30.63	12.94		-1.375	2.59		
Control	Pthr1	FAM	31.9	13.96	14.1	-0.14	1.10	1.004712	0.137452
Control	Pthr1	FAM	32.21	14.24		0.14	0.91		
PEMF	Pthr1	FAM	31.16	12.92	13.045	-1.18	2.27	2.085522	0.254906
PEMF	Pthr1	FAM	31.36	13.17		-0.93	1.91		
DM	Pthr1	FAM	30.89	13.17	13.29	-0.93	1.91	1.75928	0.20647
DM	Pthr1	FAM	31.1	13.41		-0.69	1.61		
Control	Runx	FAM	21.49	3.55	3.61	-0.056	1.04	1.001421	0.053965
Control	Runx	FAM	21.63	3.66		0.054	0.96		
PEMF	Runx	FAM	21.68	3.44	3.47	-0.166	1.12	1.099092	0.032317
PEMF	Runx	FAM	21.69	3.50		-0.106	1.08		
DM	Runx	FAM	20.55	2.83	2.985	-0.776	1.71	1.546826	0.234125
DM	Runx	FAM	20.83	3.14		-0.466	1.38		
Control	Sp7	FAM	25.85	7.91	7.9	0	1.00	1.00698	0.009871
Control	Sp7	FAM	25.86	7.89		-0.02	1.01		
PEMF	Sp7	FAM	25.25	7.01	7.14	-0.9	1.87	1.712198	0.217603
PEMF	Sp7	FAM	25.46	7.27		-0.64	1.56		
DM	Sp7	FAM	24.61	6.89	6.79	-1.02	2.03	2.178693	0.213227
DM	Sp7	FAM	24.38	6.69		-1.22	2.33		

Control	Ibsp	FAM	19.68	1.74	1.765	-0.03	1.02	1.003622	0.024593
Control	Ibsp	FAM	19.76	1.79		0.02	0.99		
PEMF	Ibsp	FAM	18.94	0.7	0.785	-1.07	2.10	1.98275	0.165016
PEMF	Ibsp	FAM	19.06	0.87		-0.9	1.87		
DM	Ibsp	FAM	17.34	-0.38	-0.395	-2.15	4.44	4.484907	0.065943
DM	Ibsp	FAM	17.28	-0.41		-2.18	4.53		

Block Type	96well
Chemistry	TAQMAN
Experiment File Name	H:\Cell Mol Med JoeD\Real-Time PCR\MC3T3 Osteocal BSPI gapdh.eds
Experiment Run End Time	2020-11-17 12:08:10 PM EDT
Instrument Type	steponeplus
Passive Reference	ROX

Sample Name	Target Name	Reporter	Cт	ΔCт	ΔCт Mean	ΔΔCт	Fold	Mean Fold	SD
Control	BSPI	FAM	18.19	0.46	0.335	0.125	0.92	1.003756	0.122686
Control	BSPI	FAM	17.94	0.21		-0.125	1.09		
PEMF	BSPI	FAM	13.9	-2.135	-2.035	-2.47	5.54	5.181835	0.507142
PEMF	BSPI	FAM	14.1	-1.935		-2.27	4.82		
DM	BSPI	FAM	14.89	-2.57	-2.645	-2.905	7.49	7.900525	0.580319
DM	BSPI	FAM	14.74	-2.72		-3.055	8.31		
Control	Osteocal	FAM	18.91	1.18	1.205	-0.025	1.02	1.00015	0.024508
Control	Osteocal	FAM	18.96	1.23		0.025	0.98		
PEMF	Osteocal	FAM	14.43	-1.605	-1.495	-2.81	7.01	6.516916	0.70135
PEMF	Osteocal	FAM	14.65	-1.385		-2.59	6.02		
DM	Osteocal	FAM	15.5	-1.96	-1.835	-3.165	8.97	8.255802	1.009078
DM	Osteocal	FAM	15.75	-1.71		-2.915	7.54		
				mean					
Control	gapdh	VIC	17.93	17.73					
Control	gapdh	VIC	17.53	17.73					
PEMF	gapdh	VIC	16.02	16.035					
PEMF	gapdh	VIC	16.05	16.035					
DM	gapdh	VIC	17.15	17.46					
DM	gapdh	VIC	17.77	17.46					

		mean							sd	
	Alpl	cFos	Pthr1	Runx	Sp7	Ibsp	BSPI	Osteocal	Alpl	c
Control	1	1	1	1	1	1	1	1	0.04	
PEMF	3.57	2.8	2.1	1.11	1.71	1.98	5.18	6.52	0.5	
DM	3.76	2.3	1.8	1.56	2.18	4.48	7.9	8.26	0.48	

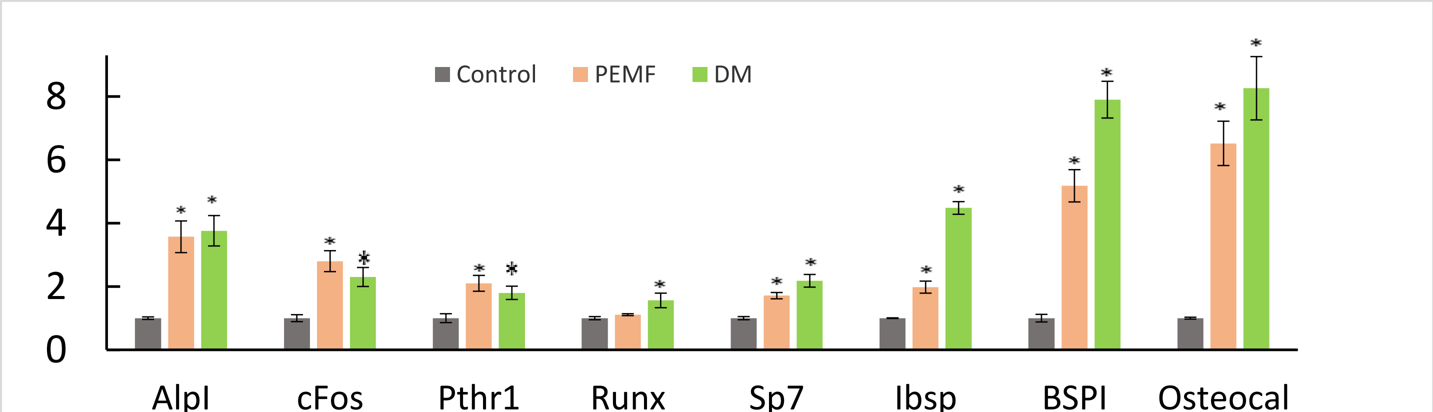

Figure 3A

Protein

0.0686	0.1124	0.1868	0.3178	0.4148	0.5356	0.7646
						40

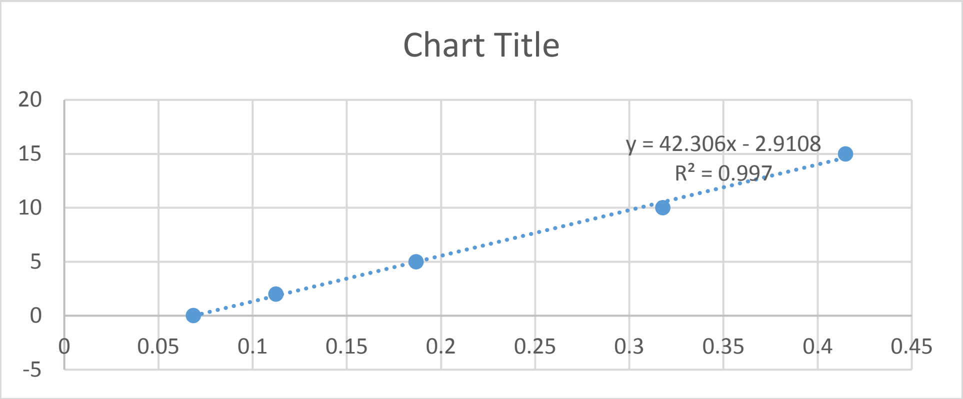

Raw Data

0.4715	0.4391	0.4292	0.4302	0.5279	0.5515	0.4139	0.3807	0.354	0.2521
0.4553		0.4297		0.5397		0.3973		0.30305	
0.4533	0.4949	0.4185	0.3756	0.5019	0.5042	0.2795	0.2773	0.3042	0.3511
0.4741		0.39705		0.50305		0.2784		0.32765	
0.4024	0.3399	0.3521	0.3714	0.3929	0.3551	0.3018	0.3717	0.2361	0.261
0.37115		0.36175		0.374		0.33675		0.24855	

Protein				mg/ml (All data)	mg/ml (exclude last two)
	(-)	11	0.455	10.1	8.2
		28	0.43	9.4	7.6
		wt	0.54	12.5	10.0
	4D	11	0.397	8.5	6.9
		28	0.303	5.8	5.0
		wt	0.332	6.6	5.6
	8D	11	0.474	10.7	8.6
		28	0.397	8.5	6.9
		wt	0.503	11.5	9.2
	12D	11	0.278	5.1	4.4
		28	0.328	6.5	5.5
		wt	0.414	9.0	7.3
	16D	11	0.371	7.7	6.4
		28	0.362	7.5	6.2
		wt	0.374	7.8	6.5
	DM 12D	11	0.337	6.8	5.7
		28	0.286	5.3	4.6
		wt	0.473	10.6	8.5

0.5051	0.4942	0.5537	0.5668	0.8632	0.749	0.5266	0.4611	0.4817	0.4032
0.49965	0.0077075	0.56025	0.009263	0.8061	0.080752	0.49385	0.046315	0.44245	0.055508
0.7967	0.5611	0.6124	0.5009	0.8061	0.7669	0.4211	0.4995	0.5246	0.5181
0.6789	0.1665944	0.55665	0.078842	0.7865	0.027719	0.4603	0.055437	0.52135	0.004596
0.7685	0.5473	0.5609	0.5972	0.854	0.5854	1.7704	2.004	0.5699	0.6457
0.6579	0.156412	0.57905	0.025668	0.7197	0.189929	1.8872	0.16518	0.6078	0.053599
0.324	0.3263								

0.32515 0.0016263

			Mean	stdev	corrected	corrected for protein	Stdev
Alk Phos	(-)	11	0.5	0.008	0.285	1.01	0.016094
		28	0.56	0.009	0.345	1.31	0.02105
		wt	0.706	0.081	0.491	1.40	0.160453
	4D	11	0.494	0.046	0.279	1.18	0.109576
		28	0.442	0.055	0.227	1.40	0.17419
		wt	0.472	0.053	0.257	1.39	0.155756
	8D	11	0.679	0.166	0.464	1.55	0.380058
		28	0.557	0.079	0.342	1.44	0.204588
		wt	0.786	0.028	0.571	1.78	0.063251
	12D	11	0.46	0.055	0.245	1.72	0.205958
		28	0.521	0.005	0.306	1.68	0.016128
		wt	0.596	0.017	0.381	1.52	0.043358
	16D	11	0.658	0.156	0.443	2.05	0.485414
		28	0.579	0.025	0.364	1.74	0.075131
		wt	0.719	0.189	0.504	2.30	0.605619
	DM 12D	11	0.8	0.165	0.585	3.09	0.637506
		28	0.608	0.054	0.393	2.64	0.234877
		wt	0.906	0.019	0.691	2.32	0.048681

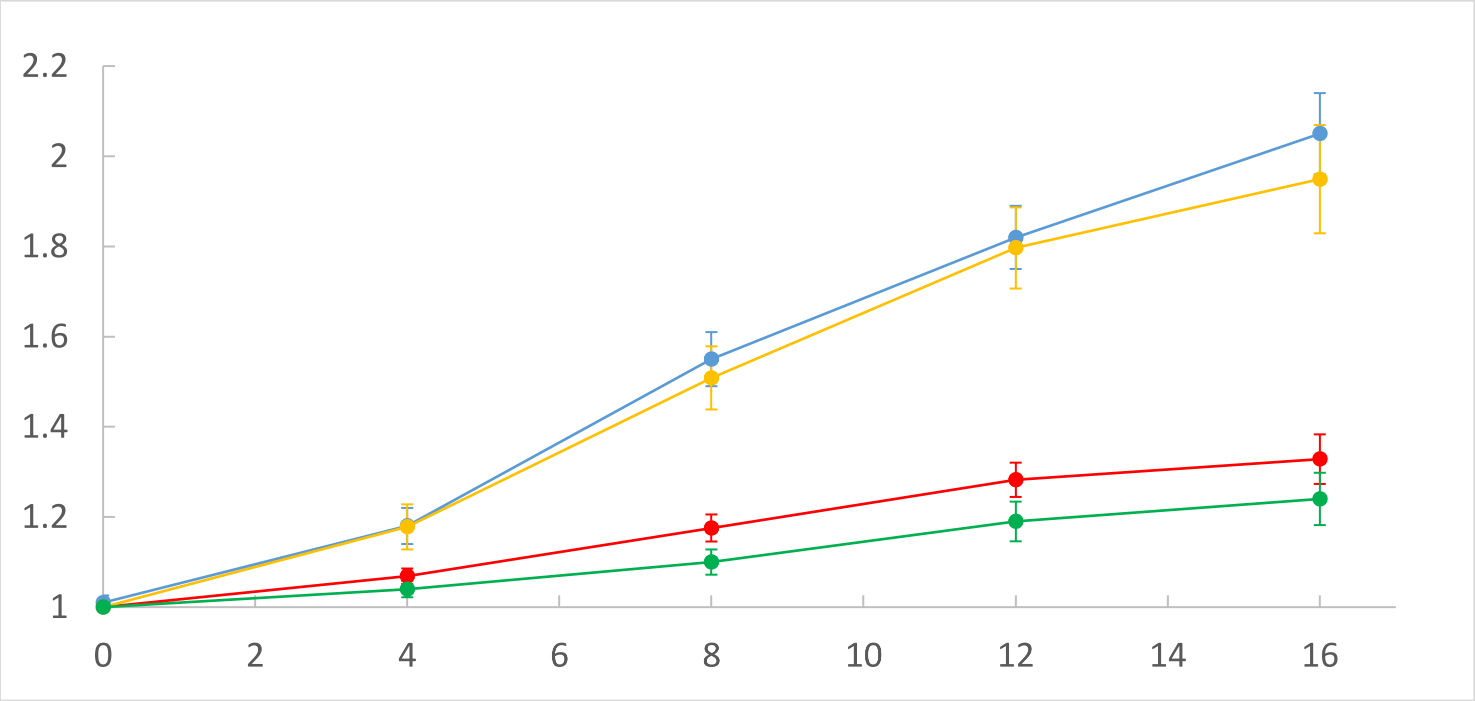

Figure 3B

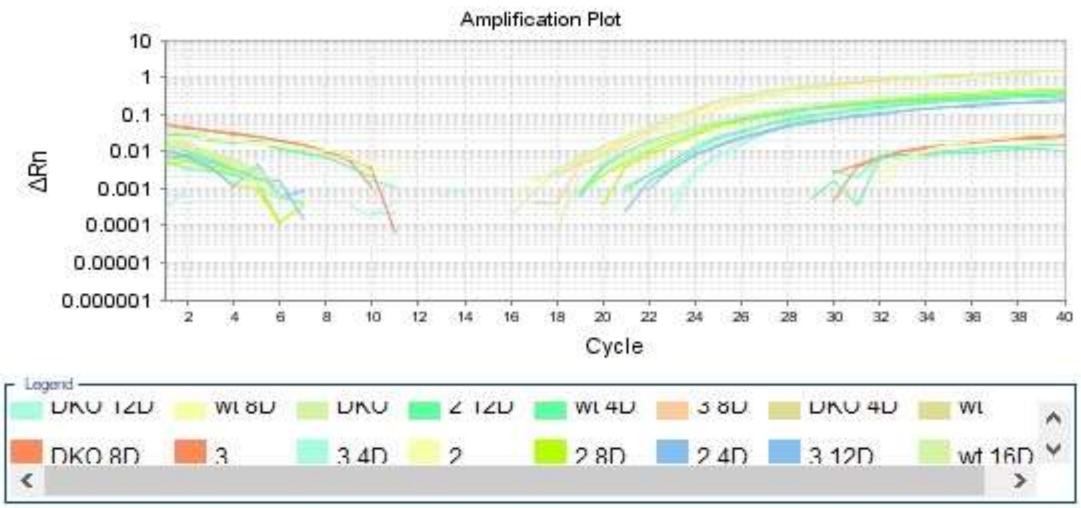

Block Type	96well
Chemistry	TAQMAN
Experiment File Name	H:\Users\Itsme\Documents\Lab\Data\Publications\Manuscript A2 A3\Real-Time PCR\MC3T3 4 8 12 gapdh
Experiment Run End Time	2020-03-31 12:35:50 PM EDT
Instrument Type	steponeplus
Passive Reference	ROX

Day	Sample Name	Target Name	Reporter	Cт	Cт Mean	ΔCт	ΔCт Mean	ΔΔCт	Fold
0	WT	Alpl		20.84	21.04	2.62	2.82	-0.20	1.144724
	WT	Alpl		21.23		3.01	2.82	0.20	0.873573
	Δ2A	Alpl		21.53	21.786	3.32	3.58	-0.26	1.194163
	Δ2A	Alpl		22.04		3.84	3.58	0.26	0.837406
	Δ3	Alpl		21.04	20.68	3.21	2.85	0.36	0.779165
	Δ3	Alpl		20.32		2.49	2.85	-0.36	1.283426
	DKO	Alpl		20.91	20.69983	3.96	3.75	0.21	0.866572
	DKO	Alpl		20.49		3.55	3.75	-0.21	1.153973
4	WT	Alpl		21.02	20.86303	1.32	1.17	-1.49	2.812476
	WT	Alpl		20.70		1.01	1.17	-1.81	3.504821
	Δ2A	Alpl		22.25	22.34095	3.34	3.43	-0.24	1.182858
	Δ2A	Alpl		22.43		3.52	3.43	-0.06	1.040401
	Δ3	Alpl		20.97	20.69952	2.92	2.65	0.08	0.947978
	Δ3	Alpl		20.42		2.37	2.65	-0.47	1.38781
	DKO	Alpl		20.05	20.155	2.29	2.39	-1.47	2.767102
	DKO	Alpl		20.26		2.50	2.39	-1.26	2.392263
8	WT	Alpl		18.42	18.57321	0.75	0.91	-2.07	4.185645
	WT	Alpl		18.73		1.06	0.91	-1.75	3.367935
	Δ2A	Alpl		22.07	22.35296	2.66	2.94	-0.92	1.893993
	Δ2A	Alpl		22.64		3.22	2.94	-0.36	1.280489
	Δ3	Alpl		19.66	19.84791	2.44	2.63	-0.41	1.328299
	Δ3	Alpl		20.04		2.82	2.87	-0.03	1.019272
	DKO	Alpl		19.74	20.0316	2.93	3.22	-0.83	1.77429
	DKO	Alpl		20.32		3.50	1.96	-0.25	1.189573
12	WT	Alpl		17.96	18.04	0.42	0.50	-2.40	5.263259
	WT	Alpl		18.12		0.58	1.43	-2.24	4.710748

		Δ2A	Alpl	21.47	21.32356	2.28	2.13	-1.30	2.458395	[illegible]
		Δ2A	Alpl	21.18		1.98	2.23	-1.59	3.020549	
		Δ3	Alpl	22.56	22.35	2.48	2.27	-0.36	1.286807	
		Δ3	Alpl	22.14		2.06	2.26	-0.78	1.721654	
		DKO	Alpl	19.85		2.46	2.56	-1.30	2.458833	[illegible]
		DKO	Alpl	20.05	20.41476	2.66	1.31	-1.10	2.140539	
	16	WT	Alpl	20.78		-0.04	0.06	-2.86	7.247843	
		WT	Alpl	20.97	20.25745	0.15	0.57	-2.66	6.329937	
		Δ2A	Alpl	19.54	19.655	0.98	1.10	-2.59	6.0399	[illegible]
		Δ2A	Alpl	19.77		1.22	1.56	-2.36	5.14983	
		Δ3	Alpl	20.68	20.78135	1.91	2.01	-0.94	1.919423	
		Δ3	Alpl	20.89		2.12	1.88	-0.73	1.660218	
		DKO	Alpl	20.59	20.678	1.64	1.73	-2.12	4.343511	[illegible]
		DKO	Alpl	20.77		1.82	0.84	-1.93	3.823415	
DM 16		WT	Alpl	21.06	20.96	-0.14	-0.24	-2.96	7.767734	[illegible]
		WT	Alpl	20.86		-0.34	0.14	-3.16	8.913279	
		Δ2A	Alpl	21.98	21.928	0.63	0.58	-2.95	7.724945	[illegible]
		Δ2A	Alpl	21.88		0.53	0.82	-3.05	8.302379	
		Δ3	Alpl	22.76	22.91	1.11	1.26	-1.74	3.34585	[illegible]
		Δ3	Alpl	23.06		1.41	1.51	-1.44	2.717675	
		DKO	Alpl	24.16	24.355	1.61	1.81	-2.14	4.422499	[illegible]
		DKO	Alpl	24.55		2.00	2.00	-1.75	3.37494	
	0	WT	Gapdh	17.98	18.21904					
		WT	Gapdh	18.46	18.22					
		Δ2A	Gapdh	18.47	18.21					
		Δ2A	Gapdh	17.95	18.21					
		Δ3	Gapdh	17.82	17.83263					
		Δ3	Gapdh	17.85	17.83					
		DKO	Gapdh	17.43	16.94497					
		DKO	Gapdh	16.46	16.94					
	4	WT	Gapdh	19.73	19.69767					
		WT	Gapdh	19.67	19.70					
		Δ2A	Gapdh	18.89	18.91113					
		Δ2A	Gapdh	18.93	18.91					
		Δ3	Gapdh	18.05	18.05001					
		Δ3	Gapdh	18.05	18.05					
		DKO	Gapdh	18.00	17.76351					
		DKO	Gapdh	17.53	17.76					
	8	WT	Gapdh	17.71	17.66591					
		WT	Gapdh	17.62	17.67					
		Δ2A	Gapdh	19.62	19.4125					
		Δ2A	Gapdh	19.20	19.41					
		Δ3	Gapdh	17.01	17.2191					
		Δ3	Gapdh	17.43	17.22					
		DKO	Gapdh	16.88	16.81558					
		DKO	Gapdh	16.75	16.82					
	12	WT	Gapdh	17.05	17.54					
		WT	Gapdh	18.03	17.54					
		Δ2A	Gapdh	19.05	19.1903					
		Δ2A	Gapdh	19.33	19.19					

		Δ3	Gapdh	20.01	20.07642
		Δ3	Gapdh	20.14	20.08
		DKO		17.28	17.39311
		DKO	Gapdh	17.51	17.39
	16	WT	Gapdh	20.68	20.82113
		WT	Gapdh	20.96	20.82
		Δ2A	Gapdh	19.06	18.555
		Δ2A	Gapdh	18.05	18.56
		Δ3	Gapdh	19.04	18.77
		Δ3	Gapdh	18.50	18.77
		DKO	Gapdh	19.30	18.95
		DKO	Gapdh	18.60	18.95
DM 16		WT	Gapdh	20.10	21.2
		WT	Gapdh	22.30	21.20
		Δ2A	Gapdh	21.40	21.35
		Δ2A	Gapdh	21.30	21.35
		Δ3	Gapdh	21.76	21.655
		Δ3	Gapdh	21.55	21.66
		DKO	Gapdh	22.50	22.55
		DKO	Gapdh	22.60	22.55

		WT	∆2A	∆3	DKO
Mean					
	0		1	1	1
	4		1.11	1.17	1.29
	8		1.56	1.17	1.48
	12		2.74	1.5	2.3
	16		5.59	1.79	4.08
	DM 16		8.01	3.03	3.9
STDEV	0		0.25	0.36	0.2
	4		0.11	0.31	0.13
	8		0.43	0.22	0.42
	12		0.4	0.31	0.22
	16		0.63	0.18	0.37
	DM 16		0.43	0.44	0.74

Figure 4 B, C

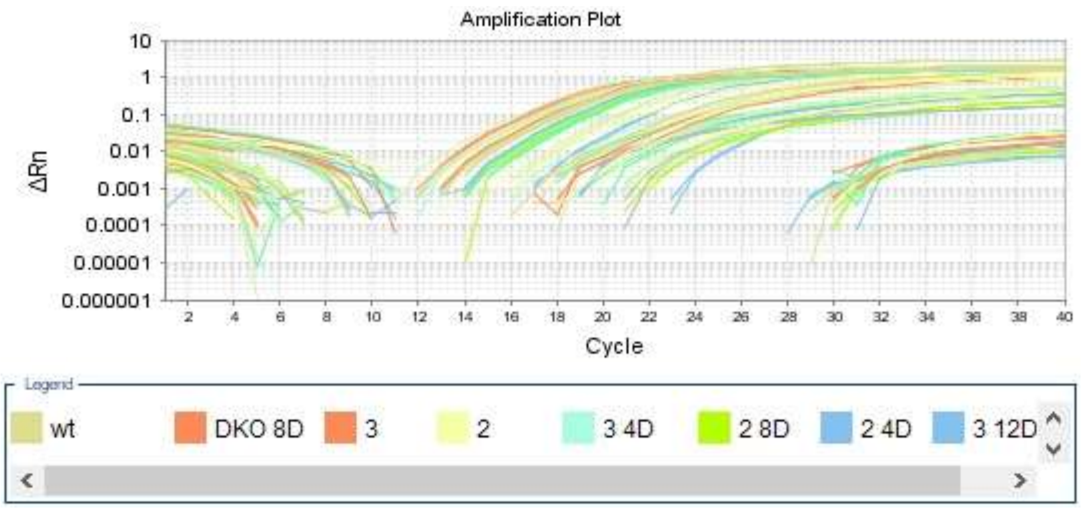

Figure 4 A, D, E

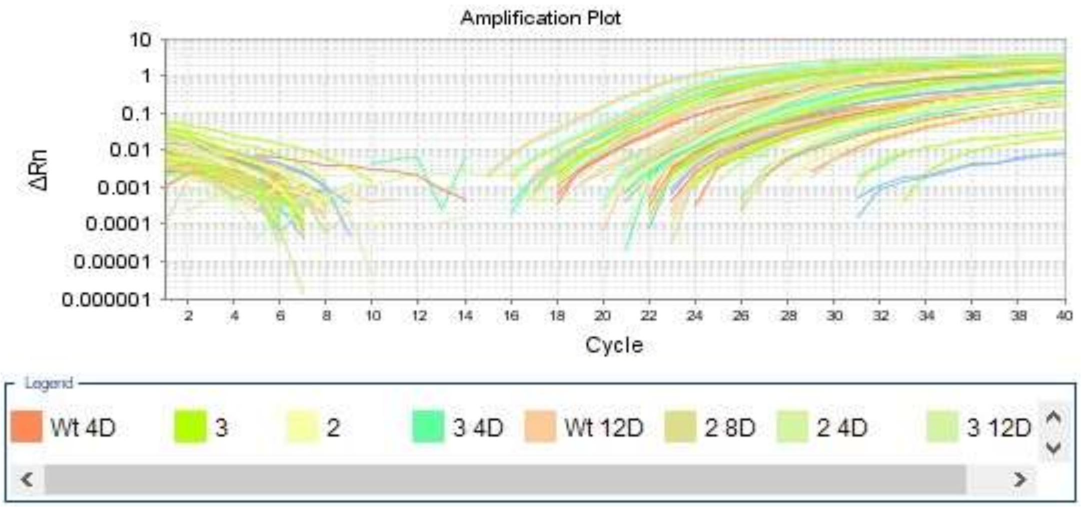

Block Type	96well
Chemistry	TAQMAN
Experiment File Name	H:\Users\Itsme\Documents\Lab\Data\Publications\Manuscript A2 A3\Real-Time PCR\MC3T3 4 8 12 gapdh
Experiment Run End Time	2020-03-31 12:35:50 PM EDT
Instrument Type	steponeplus
Passive Reference	ROX

Day	Sample Name	Target Name	Cт	Cт Mean	ΔCт	ΔCт Mean	ΔΔCт	Fold	Mea Fol
0	WT	cFos	26.84	27.04	8.62	8.62	0.00	1.00	
	WT	cFos	27.23			8.62			
	Δ2A	cFos	26.83	26.886	8.62	8.68	0.00	1.00	
	Δ2A	cFos	26.94		8.74	8.68	0.12	0.92	
	Δ3	cFos	27.24	27.03	9.41	9.20	0.79	0.58	
	Δ3	cFos	26.82		8.99	9.20	0.37	0.78	
4	WT	cFos	29.02	28.86	9.32	9.16	0.70	0.61	
	WT	cFos	28.70		9.00	9.16	0.38	0.77	
	Δ2A	cFos	28.73	28.82	9.82	9.91	1.14	0.45	
	Δ2A	cFos	28.91		10.00	9.91	1.32	0.40	
	Δ3	cFos	27.14	26.965	9.09	8.91	-0.11	1.08	
	Δ3	cFos	26.79		8.74	8.91	-0.46	1.37	
8	WT	cFos	25.02	25.09	7.35	7.42	-1.27	2.41	
	WT	cFos	25.16		7.49	7.42	-1.13	2.18	
	Δ2A	cFos	28.07	28.19	8.66	8.78	-0.02	1.02	
	Δ2A	cFos	28.31		8.90	8.78	0.22	0.86	
	Δ3	cFos	25.35	25.245	8.13	8.03	-1.07	2.09	
	Δ3	cFos	25.14		7.92	7.92	-1.28	2.42	
12	WT	cFos	23.18	23.1	5.64	5.56	-2.98	7.90	
	WT	cFos	23.02		5.48	7.20	-3.14	8.82	
	Δ2A	cFos	28.12	28.23	8.93	9.04	0.25	0.84	
	Δ2A	cFos	28.34		9.15	7.82	0.47	0.72	
	Δ3	cFos	26.56	26.65	6.48	6.57	-2.71	6.56	
	Δ3	cFos	26.74		6.66	6.66	-2.53	5.79	
16	WT	cFos	26.73		5.91	5.79	-2.71	6.54	
	WT	cFos	26.49	27.1135	5.67	7.43	-2.95	7.75	
	Δ2A	cFos	27.74	27.795	9.19	9.24	0.51	0.70	
	Δ2A	cFos	27.85		9.30	8.10	0.62	0.65	
	Δ3	cFos	25.67	25.765	6.90	7.00	-2.30	4.92	
	Δ3	cFos	25.86		7.09	7.09	-2.11	4.31	
DM 16	WT	cFos	27.84	27.94	6.64	6.74	-1.98	3.95	
	WT	cFos	28.03		6.83	7.18	-1.79	3.46	
	Δ2A	cFos	28.88	28.77	7.53	7.42	-1.15	2.22	
	Δ2A	cFos	28.66		7.31	7.21	-1.37	2.58	
	Δ3	cFos	28.76	28.855	7.11	7.20	-2.09	4.26	
	Δ3	cFos	28.95		7.30	7.30	-1.90	3.74	
0	WT	Gapdh	17.98	18.21904					
	WT	Gapdh	18.46	18.22					
	Δ2A	Gapdh	18.47	18.21					
	Δ2A	Gapdh	17.95	18.21					
	Δ3	Gapdh	17.82	17.83263					

	Δ3	Gapdh	17.85	17.83
4	WT	Gapdh	19.73	19.69767
	WT	Gapdh	19.67	19.70
	Δ2A	Gapdh	18.89	18.91113
	Δ2A	Gapdh	18.93	18.91
	Δ3	Gapdh	18.05	18.05001
	Δ3	Gapdh	18.05	18.05
8	WT	Gapdh	17.71	17.66591
	WT	Gapdh	17.62	17.67
	Δ2A	Gapdh	19.62	19.4125
	Δ2A	Gapdh	19.20	19.41
	Δ3	Gapdh	17.01	17.2191
	Δ3	Gapdh	17.43	17.22
12	WT	Gapdh	17.05	17.54
	WT	Gapdh	18.03	17.54
	Δ2A	Gapdh	19.05	19.1903
	Δ2A	Gapdh	19.33	19.19
	Δ3	Gapdh	20.01	20.07642
	Δ3	Gapdh	20.14	20.08
16	WT	Gapdh	20.68	20.82113
	WT	Gapdh	20.96	20.82
	Δ2A	Gapdh	19.06	18.555
	Δ2A	Gapdh	18.05	18.56
	Δ3	Gapdh	19.04	18.77
	Δ3	Gapdh	18.50	18.77
DM 16	WT	Gapdh	20.10	21.2
	WT	Gapdh	22.30	21.20
	Δ2A	Gapdh	21.40	21.35
	Δ2A	Gapdh	21.30	21.35
	Δ3	Gapdh	21.76	21.655
	Δ3	Gapdh	21.55	21.66
		Mean		SD

cFos	WT	2A	3	WT	2A	3
Control	1	0.96	0.68	0.1	0.05	0.14
4d PEMF	0.69	0.74	1.23	0.11	0.07	0.21
8d PEMF	2.3	1.63	2.26	0.16	0.19	0.23
12d PEMF	8.36	1.36	6.18	0.65	0.15	0.54
16d PEMF	7.15	1.18	4.61	0.85	0.06	0.43
Differentiation Media	3.7	4.18	4	0.35	0.45	0.37

Day	Sample Name	Target Name	Cт	Cт Mean	ΔCт	ΔCт Mean	ΔΔCт	Fold	Mea Fol
0	WT	Ibsp	26.04	26.62	7.82	7.82	0.00	1.00	
	WT	Ibsp	27.20			7.82			
	Δ2A	Ibsp	25.78	25.92	7.57	7.71	-0.25	1.19	
	Δ2A	Ibsp	26.06		7.85	7.71	0.03	0.98	
	Δ3	Ibsp	26.00	26.41	8.17	8.58	0.35	0.79	
	Δ3	Ibsp	26.82		8.99	8.58	1.17	0.45	
4	WT	Ibsp	27.62	27.545	7.92	7.85	0.10	0.93	
	WT	Ibsp	27.47		7.77	7.85	-0.05	1.03	
	Δ2A	Ibsp	26.43	26.32	7.52	7.41	-0.19	1.14	
	Δ2A	Ibsp	26.21		7.30	7.41	-0.41	1.33	
	Δ3	Ibsp	26.44	26.565	8.39	8.51	-0.19	1.14	
	Δ3	Ibsp	26.69		8.64	8.51	0.06	0.96	
8	WT	Ibsp	24.52	24.59	6.85	6.92	-0.97	1.95	
	WT	Ibsp	24.66		6.99	6.92	-0.83	1.77	
	Δ2A	Ibsp	26.17	26.04	6.76	6.63	-0.96	1.94	
	Δ2A	Ibsp	25.91		6.50	6.63	-1.22	2.32	
	Δ3	Ibsp	25.65	25.595	8.43	8.38	-0.15	1.11	
	Δ3	Ibsp	25.54		8.32	8.32	-0.26	1.19	
12	WT	Ibsp	23.48	23.55	5.94	6.01	-1.88	3.68	
	WT	Ibsp	23.62		6.08	5.99	-1.74	3.34	
	Δ2A	Ibsp	25.10	24.97	5.91	5.78	-1.80	3.49	
	Δ2A	Ibsp	24.84		5.65	7.07	-2.06	4.18	
	Δ3	Ibsp	28.56	28.45	8.48	8.37	-0.09	1.07	
	Δ3	Ibsp	28.34		8.26	8.26	-0.31	1.24	
16	WT	Ibsp	26.73		5.91	6.04	-1.91	3.76	

		WT	Ibsp		26.99	25.8635	6.17	6.18	-1.65	3.15
		Δ2A	Ibsp		24.74	24.645	6.19	6.09	-1.53	2.88
		Δ2A	Ibsp		24.55		6.00	7.20	-1.72	3.29
		Δ3	Ibsp		27.17	27.065	8.40	8.30	-0.18	1.13
		Δ3	Ibsp		26.96		8.19	8.19	-0.39	1.31
DM 16		WT	Ibsp		27.16	27.10	5.96	5.90	-1.86	3.63
		WT	Ibsp		27.03		5.83	5.78	-1.99	3.97
		Δ2A	Ibsp		27.08	26.97	5.73	5.62	-1.98	3.95
		Δ2A	Ibsp		26.86		5.51	6.81	-2.20	4.61
		Δ3	Ibsp		29.76	29.905	8.11	8.25	-0.47	1.39
		Δ3	Ibsp		30.05		8.40	8.40	-0.18	1.13
	0	WT	Gapdh		17.98	18.21904				
		WT	Gapdh		18.46	18.22				
		Δ2A	Gapdh		18.47	18.21				
		Δ2A	Gapdh		17.95	18.21				
		Δ3	Gapdh		17.82	17.83263				
		Δ3	Gapdh		17.85	17.83				
	4	WT	Gapdh		19.73	19.69767				
		WT	Gapdh		19.67	19.70				
		Δ2A	Gapdh		18.89	18.91113				
		Δ2A	Gapdh		18.93	18.91				
		Δ3	Gapdh		18.05	18.05001				
		Δ3	Gapdh		18.05	18.05				
	8	WT	Gapdh		17.71	17.66591				
		WT	Gapdh		17.62	17.67				
		Δ2A	Gapdh		19.62	19.4125				
		Δ2A	Gapdh		19.20	19.41				
		Δ3	Gapdh		17.01	17.2191				
		Δ3	Gapdh		17.43	17.22				
	12	WT	Gapdh		17.05	17.54				
		WT	Gapdh		18.03	17.54				
		Δ2A	Gapdh		19.05	19.1903				
		Δ2A	Gapdh		19.33	19.19				
		Δ3	Gapdh		20.01	20.07642				
		Δ3	Gapdh		20.14	20.08				
	16	WT	Gapdh		20.68	20.82113				
		WT	Gapdh		20.96	20.82				
		Δ2A	Gapdh		19.06	18.555				
		Δ2A	Gapdh		18.05	18.56				
		Δ3	Gapdh		19.04	18.77				
		Δ3	Gapdh		18.50	18.77				
DM 16		WT	Gapdh		20.10	21.2				
		WT	Gapdh		22.30	21.20				
		Δ2A	Gapdh		21.40	21.35				
		Δ2A	Gapdh		21.30	21.35				
		Δ3	Gapdh		21.76	21.655				
		Δ3	Gapdh		21.55	21.66				
			Mean			SD				
Ibsp		WT	2A	3	WT	2A	3			

Control	1	1.08	0.62	0.1	0.15	0.24
4d PEMF	0.98	1.24	1.05	0.07	0.13	0.13
8d PEMF	1.86	2.13	1.15	0.13	0.23	0.06
12d PEMF	3.51	3.84	1.16	0.24	0.49	0.12
16d PEMF	3.45	3.09	1.22	0.43	0.29	0.13
Differentiation Media	3.8	4.23	1.26	0.24	0.46	0.18

Day	Sample Name	Target Name	C_T	C_T Mean	ΔC_T	ΔC_T Mean	$\Delta\Delta C_T$	Fold	Mea Fol
0	WT	Pthr1	28.84	29.04	10.62	10.62	0.00	1.00	
	WT	Pthr1	29.23			10.62			
	Δ2A	Pthr1	28.93	29.086	10.72	10.88	0.10	0.93	
	Δ2A	Pthr1	29.24		11.04	10.88	0.42	0.75	
	Δ3	Pthr1	28.64	28.78	10.81	10.95	0.19	0.88	
	Δ3	Pthr1	28.92		11.09	10.95	0.47	0.72	
4	WT	Pthr1	30.62	30.545	10.92	10.85	0.30	0.81	
	WT	Pthr1	30.47		10.77	10.85	0.15	0.90	
	Δ2A	Pthr1	30.23	30.12	11.32	11.21	0.44	0.74	
	Δ2A	Pthr1	30.01		11.10	11.21	0.22	0.86	
	Δ3	Pthr1	29.14	29.265	11.09	11.21	0.14	0.91	
	Δ3	Pthr1	29.39		11.34	11.21	0.39	0.76	
8	WT	Pthr1	27.32	27.24	9.65	9.57	-0.97	1.95	
	WT	Pthr1	27.16		9.49	9.57	-1.13	2.18	
	Δ2A	Pthr1	30.07	30.19	10.66	10.78	-0.22	1.17	
	Δ2A	Pthr1	30.31		10.90	10.78	0.02	0.99	
	Δ3	Pthr1	27.35	27.245	10.13	10.03	-0.82	1.76	
	Δ3	Pthr1	27.14		9.92	9.92	-1.03	2.04	
12	WT	Pthr1	26.08	26.15	8.54	8.61	-2.08	4.23	
	WT	Pthr1	26.22		8.68	9.80	-1.94	3.84	
	Δ2A	Pthr1	30.12	30.185	10.93	10.99	0.05	0.97	
	Δ2A	Pthr1	30.25		11.06	10.01	0.18	0.88	
	Δ3	Pthr1	29.03	29.135	8.95	9.06	-1.99	3.98	
	Δ3	Pthr1	29.24		9.16	9.16	-1.78	3.44	
16	WT	Pthr1	29.33		8.51	8.44	-2.11	4.32	

		WT	Pthr1		29.19	29.4635	8.37	9.78	-2.25	4.77
		Δ2A	Pthr1		29.74	29.645	11.19	11.09	0.31	0.81
		Δ2A	Pthr1		29.55		11.00	9.95	0.12	0.92
		Δ3	Pthr1		27.67	27.765	8.90	9.00	-2.05	4.13
		Δ3	Pthr1		27.86		9.09	9.09	-1.86	3.62
DM 16		WT	Pthr1		30.14	30.06	8.94	8.86	-1.68	3.21
		WT	Pthr1		29.98		8.78	9.38	-1.84	3.58
		Δ2A	Pthr1		31.33	31.495	9.98	10.15	-0.90	1.87
		Δ2A	Pthr1		31.66		10.31	10.06	-0.57	1.48
		Δ3	Pthr1		31.46	31.36	9.81	9.71	-1.14	2.21
		Δ3	Pthr1		31.26		9.61	9.61	-1.34	2.54
	0	WT	Gapdh		17.98	18.21904				
		WT	Gapdh		18.46	18.22				
		Δ2A	Gapdh		18.47	18.21				
		Δ2A	Gapdh		17.95	18.21				
		Δ3	Gapdh		17.82	17.83263				
		Δ3	Gapdh		17.85	17.83				
	4	WT	Gapdh		19.73	19.69767				
		WT	Gapdh		19.67	19.70				
		Δ2A	Gapdh		18.89	18.91113				
		Δ2A	Gapdh		18.93	18.91				
		Δ3	Gapdh		18.05	18.05001				
		Δ3	Gapdh		18.05	18.05				
	8	WT	Gapdh		17.71	17.66591				
		WT	Gapdh		17.62	17.67				
		Δ2A	Gapdh		19.62	19.4125				
		Δ2A	Gapdh		19.20	19.41				
		Δ3	Gapdh		17.01	17.2191				
		Δ3	Gapdh		17.43	17.22				
	12	WT	Gapdh		17.05	17.54				
		WT	Gapdh		18.03	17.54				
		Δ2A	Gapdh		19.05	19.1903				
		Δ2A	Gapdh		19.33	19.19				
		Δ3	Gapdh		20.01	20.07642				
		Δ3	Gapdh		20.14	20.08				
	16	WT	Gapdh		20.68	20.82113				
		WT	Gapdh		20.96	20.82				
		Δ2A	Gapdh		19.06	18.555				
		Δ2A	Gapdh		18.05	18.56				
		Δ3	Gapdh		19.04	18.77				
		Δ3	Gapdh		18.50	18.77				
DM 16		WT	Gapdh		20.10	21.2				
		WT	Gapdh		22.30	21.20				
		Δ2A	Gapdh		21.40	21.35				
		Δ2A	Gapdh		21.30	21.35				
		Δ3	Gapdh		21.76	21.655				
		Δ3	Gapdh		21.55	21.66				
			Mean			SD				
Pthr1		WT	2A	3	WT	2A	3			

Control	1	0.84	0.8	0.1	0.16	0.11
4d PEMF	0.86	0.8	0.83	0.06	0.09	0.1
8d PEMF	2.07	1.08	1.6	0.16	0.13	0.28
12d PEMF	4.03	0.92	3.71	0.28	0.06	0.38
16d PEMF	4.55	0.87	3.88	0.32	0.08	0.36
Differentiation Media	3.39	1.67	2.37	0.26	0.27	0.23

Day	Sample Name	Target Name	Cт	Cт Mean	ΔCт	ΔCт Mean	ΔΔCт	Fold	Mea Fol[illegible]
0	WT	Runx2	26.55	26.44	8.33	8.33	0.00	1.00	
	WT	Runx2	26.33			8.33			
	Δ2A	Runx2	27.03	26.84	8.82	8.63	0.49	0.71	
	Δ2A	Runx2	26.65		8.44	8.63	0.11	0.92	
	Δ3	Runx2	26.14	26.08	8.31	8.25	-0.02	1.02	
	Δ3	Runx2	26.02		8.19	8.25	-0.14	1.10	
4	WT	Runx2	27.89	27.78	8.19	8.08	-0.14	1.10	
	WT	Runx2	27.67		7.97	8.08	-0.36	1.28	
	Δ2A	Runx2	28.23	28.04	9.32	9.13	0.69	0.62	
	Δ2A	Runx2	27.85		8.94	9.13	0.31	0.81	
	Δ3	Runx2	26.21	26.32	8.16	8.27	-0.09	1.06	
	Δ3	Runx2	26.43		8.38	8.27	0.13	0.91	
8	WT	Runx2	25.52	25.64	7.85	7.97	-0.48	1.39	
	WT	Runx2	25.76		8.09	7.97	-0.24	1.18	
	Δ2A	Runx2	27.47	27.59	8.06	8.18	-0.58	1.49	
	Δ2A	Runx2	27.71		8.30	8.18	-0.34	1.26	
	Δ3	Runx2	25.35	25.245	8.13	8.03	-0.12	1.08	
	Δ3	Runx2	25.14		7.92	7.92	-0.33	1.25	
12	WT	Runx2	25.08	24.99	7.54	7.45	-0.79	1.73	
	WT	Runx2	24.90		7.36	7.64	-0.97	1.96	
	Δ2A	Runx2	27.12	27.235	7.93	8.04	-0.70	1.63	
	Δ2A	Runx2	27.35		8.16	7.97	-0.47	1.39	
	Δ3	Runx2	27.86	27.7517	7.79	7.68	-0.46	1.38	

		Δ3	Runx2	27.64		7.56	7.56	-0.68	1.61
	16	WT	Runx2	28.23		7.41	7.33	-0.92	1.89
		WT	Runx2	28.08	27.41	7.26	7.72	-1.07	2.10
		Δ2A	Runx2	26.74	26.66	8.19	8.11	-0.45	1.36
		Δ2A	Runx2	26.58		8.03	7.61	-0.61	1.52
		Δ3	Runx2	25.97	26.115	7.20	7.35	-1.05	2.07
		Δ3	Runx2	26.26		7.49	7.49	-0.76	1.69
DM 16		WT	Runx2	28.59	28.49	7.39	7.29	-0.94	1.92
		WT	Runx2	28.38		7.18	7.57	-1.15	2.22
		Δ2A	Runx2	29.30	29.49	7.95	8.14	-0.68	1.61
		Δ2A	Runx2	29.68		8.33	7.72	-0.30	1.23
		Δ3	Runx2	28.76	28.865	7.11	7.21	-1.14	2.21
		Δ3	Runx2	28.97		7.32	7.32	-0.93	1.91
	0	WT	Gapdh	17.98	18.21904				
		WT	Gapdh	18.46	18.22				
		Δ2A	Gapdh	18.47	18.21				
		Δ2A	Gapdh	17.95	18.21				
		Δ3	Gapdh	17.82	17.83263				
		Δ3	Gapdh	17.85	17.83				
	4	WT	Gapdh	19.73	19.69767				
		WT	Gapdh	19.67	19.70				
		Δ2A	Gapdh	18.89	18.91113				
		Δ2A	Gapdh	18.93	18.91				
		Δ3	Gapdh	18.05	18.05001				
		Δ3	Gapdh	18.05	18.05				
	8	WT	Gapdh	17.71	17.66591				
		WT	Gapdh	17.62	17.67				
		Δ2A	Gapdh	19.62	19.4125				
		Δ2A	Gapdh	19.20	19.41				
		Δ3	Gapdh	17.01	17.2191				
		Δ3	Gapdh	17.43	17.22				
	12	WT	Gapdh	17.05	17.54				
		WT	Gapdh	18.03	17.54				
		Δ2A	Gapdh	19.05	19.1903				
		Δ2A	Gapdh	19.33	19.19				
		Δ3	Gapdh	20.01	20.07642				
		Δ3	Gapdh	20.14	20.08				
	16	WT	Gapdh	20.68	20.82113				
		WT	Gapdh	20.96	20.82				
		Δ2A	Gapdh	19.06	18.555				
		Δ2A	Gapdh	18.05	18.56				
		Δ3	Gapdh	19.04	18.77				
		Δ3	Gapdh	18.50	18.77				
DM 16		WT	Gapdh	20.10	21.2				
		WT	Gapdh	22.30	21.20				
		Δ2A	Gapdh	21.40	21.35				
		Δ2A	Gapdh	21.30	21.35				
		Δ3	Gapdh	21.76	21.655				
		Δ3	Gapdh	21.55	21.66				

		Mean			SD	
Runx2	WT	2A	3	WT	2A	3
Control	1	0.82	1.06	0.1	0.15	0.06
4d PEMF	1.19	0.72	0.99	0.13	0.13	0.11
8d PEMF	1.28	1.38	1.17	0.15	0.16	0.2
12d PEMF	1.84	1.51	1.49	0.16	0.17	0.16
16d PEMF	2	1.44	1.88	0.15	0.11	0.27
Differentiation Media	2.07	1.42	2.06	0.21	0.26	0.21

Day	Sample Name	Target Name	Cт	Cт Mean	ΔCт	ΔCт Mean	ΔΔCт	Fold	Mea Fol
0	WT	sp7	27.87	27.55	9.65	9.65	0.00	1.00	
	WT	sp7	27.23			9.65			
	Δ2A	sp7	27.53	27.386	9.32	9.18	-0.33	1.25	
	Δ2A	sp7	27.24		9.04	9.18	-0.61	1.53	
	Δ3	sp7	27.64	27.78	9.81	9.95	0.16	0.90	
	Δ3	sp7	27.92		10.09	9.95	0.44	0.74	
4	WT	sp7	29.56	29.395	9.86	9.70	0.21	0.86	
	WT	sp7	29.23		9.53	9.70	-0.12	1.08	
	Δ2A	sp7	28.08	28.205	9.17	9.29	-0.01	1.01	
	Δ2A	sp7	28.33		9.42	9.29	0.24	0.85	
	Δ3	sp7	28.54	28.41	10.49	10.36	0.54	0.69	
	Δ3	sp7	28.28		10.23	10.36	0.28	0.82	
8	WT	sp7	26.32	26.49	8.65	8.82	-1.00	2.00	
	WT	sp7	26.66		8.99	8.82	-0.66	1.58	
	Δ2A	sp7	28.07	28.25	8.66	8.84	-0.52	1.44	
	Δ2A	sp7	28.43		9.02	8.84	-0.16	1.12	
	Δ3	sp7	27.05	26.98	9.83	9.76	-0.12	1.08	
	Δ3	sp7	26.91		9.69	9.69	-0.26	1.19	
12	WT	sp7	26.06	26.19	8.52	8.65	-1.13	2.19	
	WT	sp7	26.32		8.78	8.70	-0.87	1.83	
	Δ2A	sp7	27.82	27.535	8.63	8.34	-0.55	1.46	
	Δ2A	sp7	27.25		8.06	8.71	-1.12	2.17	
	Δ3	sp7	29.43	29.335	9.35	9.26	-0.59	1.51	
	Δ3	sp7	29.24		9.16	9.16	-0.78	1.72	
16	WT	sp7	29.52		8.70	8.56	-0.95	1.93	

		WT	sp7		29.24	27.94	8.42	8.25	-1.23	2.35
		Δ2A	sp7		26.64	26.495	8.09	7.94	-1.09	2.14
		Δ2A	sp7		26.35		7.80	8.85	-1.38	2.61
		Δ3	sp7		28.67	28.565	9.90	9.80	-0.05	1.03
		Δ3	sp7		28.46		9.69	9.69	-0.26	1.20
DM 16		WT	sp7		29.78	29.62	8.58	8.42	-1.07	2.10
		WT	sp7		29.45		8.25	7.97	-1.40	2.64
		Δ2A	sp7		29.03	28.915	7.68	7.57	-1.50	2.83
		Δ2A	sp7		28.80		7.45	8.41	-1.73	3.32
		Δ3	sp7		31.02	31.15	9.37	9.50	-0.58	1.50
		Δ3	sp7		31.28		9.63	9.63	-0.32	1.25
	0	WT	Gapdh		17.98	18.21904				
		WT	Gapdh		18.46	18.22				
		Δ2A	Gapdh		18.47	18.21				
		Δ2A	Gapdh		17.95	18.21				
		Δ3	Gapdh		17.82	17.83263				
		Δ3	Gapdh		17.85	17.83				
	4	WT	Gapdh		19.73	19.69767				
		WT	Gapdh		19.67	19.70				
		Δ2A	Gapdh		18.89	18.91113				
		Δ2A	Gapdh		18.93	18.91				
		Δ3	Gapdh		18.05	18.05001				
		Δ3	Gapdh		18.05	18.05				
	8	WT	Gapdh		17.71	17.66591				
		WT	Gapdh		17.62	17.67				
		Δ2A	Gapdh		19.62	19.4125				
		Δ2A	Gapdh		19.20	19.41				
		Δ3	Gapdh		17.01	17.2191				
		Δ3	Gapdh		17.43	17.22				
	12	WT	Gapdh		17.05	17.54				
		WT	Gapdh		18.03	17.54				
		Δ2A	Gapdh		19.05	19.1903				
		Δ2A	Gapdh		19.33	19.19				
		Δ3	Gapdh		20.01	20.07642				
		Δ3	Gapdh		20.14	20.08				
	16	WT	Gapdh		20.68	20.82113				
		WT	Gapdh		20.96	20.82				
		Δ2A	Gapdh		19.06	18.555				
		Δ2A	Gapdh		18.05	18.56				
		Δ3	Gapdh		19.04	18.77				
		Δ3	Gapdh		18.50	18.77				
DM 16		WT	Gapdh		20.10	21.2				
		WT	Gapdh		22.30	21.20				
		Δ2A	Gapdh		21.40	21.35				
		Δ2A	Gapdh		21.30	21.35				
		Δ3	Gapdh		21.76	21.655				
		Δ3	Gapdh		21.55	21.66				
			Mean			SD				
sp7		WT	2A	3	WT	2A	3			

Control	1	1.39	0.82	0.14	0.2	0.11
4d PEMF	0.97	0.93	0.75	0.16	0.11	0.1
8d PEMF	1.78	1.28	1.14	0.3	0.22	0.08
12d PEMF	2.01	1.82	1.62	0.26	0.5	0.15
16d PEMF	2.14	2.37	1.11	0.29	0.34	0.11
Differentiation Media	2.37	3.07	1.37	0.38	0.35	0.17

Figure 6

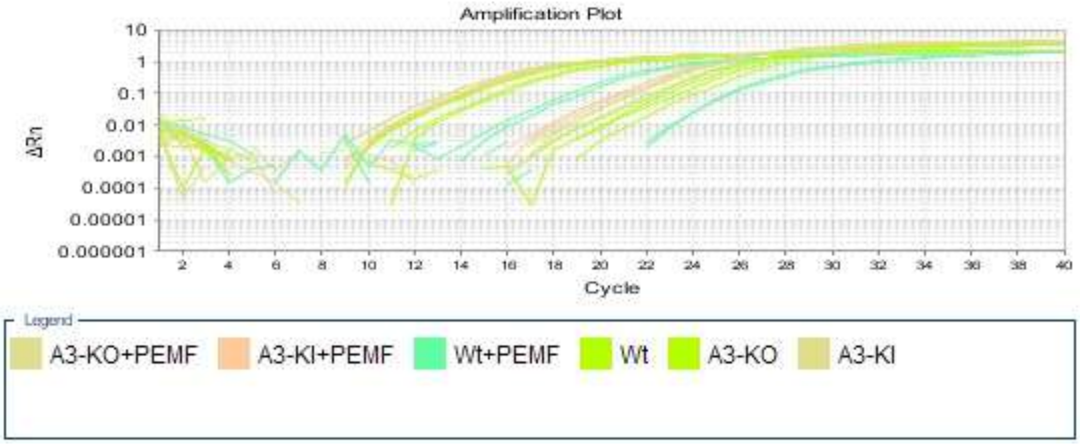

Alpl

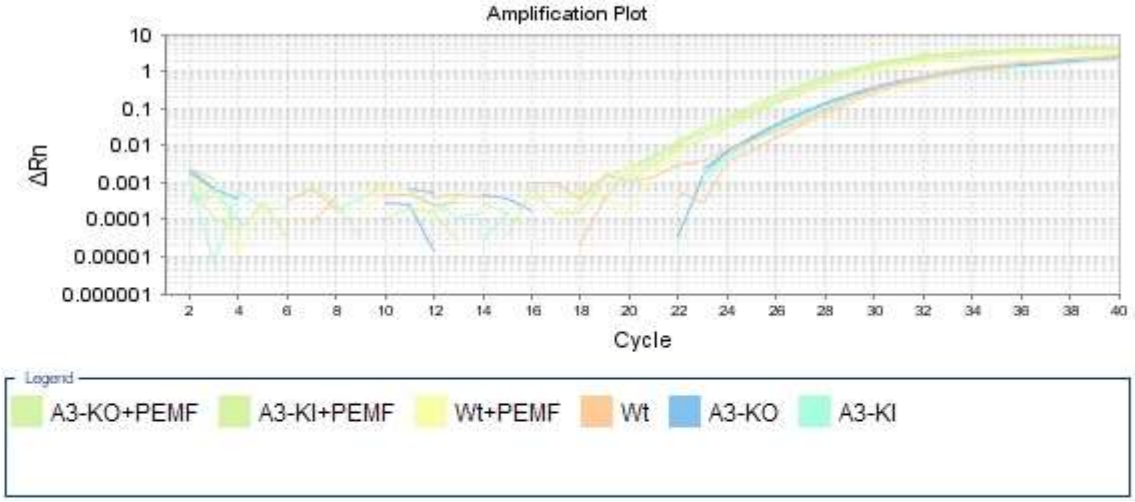

cFOS

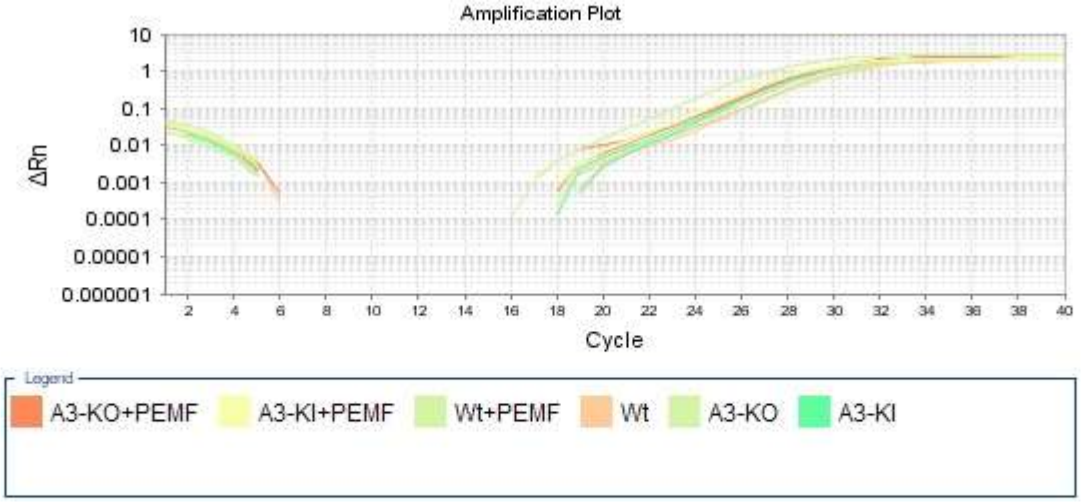

Pthr1

Block Type	96well
Chemistry	TAQMAN
Experiment File Name	H:\Cell Mol Med JoeD\Real-Time PCR\A3 KI.eds
Experiment Run End Time	2020-03-30 18:59:50 PM EDT
Instrument Type	steponeplus
Passive Reference	ROX

Sample Name	Target Name	Reporter	Cт	ΔCт	ΔCт Mean	ΔΔCт	Fold	Mean Fold	SD
WT	Alpl	FAM	21.8	8.58	8.85	-0.27	1.21	1.017564	0.266217417
WT	Alpl	FAM	22.32	9.12	8.18	0.27	0.83		
WT+PEMF	Alpl	FAM	20.68	7.24	7.1	-1.61	3.05	3.379435	0.462330335
WT+PEMF	Alpl	FAM	20.02	6.96	7.98	-1.89	3.71		
A3-KO	Alpl	FAM	22.41	9	9.195	0.15	0.90	0.794511	0.15095284
A3-KO	Alpl	FAM	22.62	9.39	8.7	0.54	0.69		
A3-KO+PEMF	Alpl	FAM	22.19	8.01	8.185	-0.84	1.79	1.597248	0.272664099
A3-KO+PEMF	Alpl	FAM	22.58	8.36	8.38	-0.49	1.40		
A3-KI	Alpl	FAM	21.81	8.4	8.565	-0.45	1.37	1.226388	0.197498738
A3-KI	Alpl	FAM	21.92	8.73	8.08	-0.12	1.09		
A3-KI+PEMF	Alpl	FAM	20.52	7.43	7.55	-1.42	2.68	2.470811	0.289975538
A3-KI+PEMF	Alpl	FAM	21.11	7.67	8.125	-1.18	2.27		
WT	cFOS	FAM	21.8	8.58	8.835	-0.285	1.22	1.037003	0.256549115
WT	cFOS	FAM	22.29	9.09	8.045	0.225	0.86		
WT+PEMF	cFOS	FAM	20.44	7	7.17	-1.865	3.64	3.260273	0.540803875
WT+PEMF	cFOS	FAM	20.4	7.34	8.19	-1.525	2.88		
A3-KO	cFOS	FAM	22.45	9.04	9.21	0.175	0.89	0.79278	0.131503891
A3-KO	cFOS	FAM	22.61	9.38	8.355	0.515	0.70		
A3-KO+PEMF	cFOS	FAM	21.51	7.33	7.445	-1.535	2.90	2.684361	0.301967854
A3-KO+PEMF	cFOS	FAM	21.78	7.56	8.025	-1.305	2.47		
A3-KI	cFOS	FAM	21.9	8.49	8.74	-0.375	1.30	1.106922	0.268584266
A3-KI	cFOS	FAM	22.18	8.99	8.185	0.125	0.92		
A3-KI+PEMF	cFOS	FAM	20.47	7.38	7.325	-1.485	2.80	2.910058	0.156817503
A3-KI+PEMF	cFOS	FAM	20.71	7.27	8.475	-1.595	3.02		
WT	Pthr1	FAM	22.9	9.68	10.05	-0.37	1.29	1.033068	0.3666846
WT	Pthr2	FAM	23.62	10.42		0.37	0.77		
WT+PEMF	Pthr3	FAM	22.28	8.84	8.7	-1.21	2.31	2.561133	0.350380874
WT+PEMF	Pthr4	FAM	21.62	8.56		-1.49	2.81		
A3-KO	Pthr5	FAM	23.4	9.99	10.23	-0.06	1.04	0.894945	0.208625588
A3-KO	Pthr6	FAM	23.7	10.47		0.42	0.75		
A3-KO+PEMF	Pthr7	FAM	22.91	8.73	8.945	-1.32	2.50	2.174919	0.455012589
A3-KO+PEMF	Pthr8	FAM	23.38	9.16		-0.89	1.85		
A3-KI	Pthr9	FAM	23.9	10.49	10.25	0.44	0.74	0.882624	0.205753378
A3-KI	Pthr10	FAM	23.2	10.01		-0.04	1.03		
A3-KI+PEMF	Pthr11	FAM	22.22	9.13	8.895	-0.92	1.89	2.256451	0.515248569
A3-KI+PEMF	Pthr12	FAM	22.1	8.66		-1.39	2.62		
WT	Gapdh	VIC	13.22						
WT	Gapdh	VIC	13.2						
WT+PEMF	Gapdh	VIC	13.44						
WT+PEMF	Gapdh	VIC	13.06						
A3-KO	Gapdh	VIC	13.41						
A3-KO	Gapdh	VIC	13.23						
A3-KO+PEMF	Gapdh	VIC	14.18						
A3-KO+PEMF	Gapdh	VIC	14.22						
A3-KI	Gapdh	VIC	13.41						
A3-KI	Gapdh	VIC	13.19						
A3-KI+PEMF	Gapdh	VIC	13.09						
A3-KI+PEMF	Gapdh	VIC	13.44						

Analysis Type	Singleplex
Endogenous Control	GAPDH
RQ Min/Max Confidence Level	95.0
Reference Sample	WT

	Fold Alpl	Fold cFOS	Fold Pthr1	SD Alpl	SD cFos	SD Pthr1
Control	1.02	1.04	1.03	0.27	0.26	0.36
Control + PEMF	3.38	3.26	2.56	0.46	0.54	0.35
A3-KO	0.79	0.79	0.89	0.15	0.13	0.21
A3-KO + PEMF	1.59	2.68	2.17	0.27	0.3	0.45
A3-KI	1.23	1.11	0.88	0.2	0.27	0.21
A3-KI + PEMF	2.47	2.91	2.26	0.29	0.17	0.51
