# Supplementary material for: Pulsed-electromagnetic-field induced osteoblast differentiation requires activation of genes downstream of adenosine receptors A2A and A3
Source: PLoS One. 2021 Feb 25;16(2):e0247659. doi: 10.1371/journal.pone.0247659 (PMC7906300; doi:10.1371/journal.pone.0247659)
Supplement: Attachment — Submitted filename: Reviewer Letter_DiDonato_012220-Revision_PONE-D-20-20625.doc [file pone.0247659.s003.doc]

Lerner Research Institute

***Joseph A. DiDonato, Ph.D.***

*Staff Scientist*

*Laboratory Director, Center for Cardiovascular Diagnostics & Prevention*

Assistant *Professor of Molecular Medicine,*

January 22, 2021	*Cleveland Clinic Lerner College of Medicine of*

*Case Western Reserve University*

*Department of Cardiovascular & Metabolic Sciences*

*Office: 216/445-2174*

*Fax: 216/636-0392*

*E-mail: didonaj@ccf.org*

Gianpaolo Papaccio, M.D., Ph.D.

PLOS ONE

Academic Editor

Re: Manuscript revision to PONE-D-20-20625

Dear Editor,

We would like to thank you and the reviewers for a constructive review of our manuscript submitted to PLOS ONE. It is our pleasure to resubmit this revised version addressing all of the points raised during the review process.

The funder, Orthofix provided support in the form of salaries for authors NZ, EIW, and JTR, during the study, but did not have any additional role in the study design, data collection and analysis, decision to publish, or preparation of the manuscript. The specific roles of these authors are articulated in the ‘author contributions’ section.

The revised manuscript includes reorganization of the original data, Results and Discussion, and has now been supplemented with new experiments that were asked for to address technical solidity and clarity in results presentation.

More specifically these were the key steps taken to address each of the reviewers’ comments point by point:

Reviewer 1

- As suggested, the Results section has been rewritten in a more detached way, and the irrelevant materials have been moved to the Discussion section as requested.
- Time-frames for each experiment are now explicitly mentioned in the Methods sections, and, together with graph colors scheme, made more consistent amongst the different panels/figures.
- Protein expression as depicted by Western Blot, as a confirmatory data for RT-PCR data, have now been included in the new Figure 5 as requested by the reviewer.

Reviewer 2

- Single time-frames in the initial submission’s experiments were exploratory in nature and later were expanded as a time-series (dose response) over a wider temporal range. As mentioned above for Reviewer 1’s request, in the revised version of the manuscript, each of the experimental time-points were made consistent among the different panels/figures.
- Both messenger RNA (Figure 1B) and protein expression (Figure 5) were included for the mature osteoblast markers, Osteocalcin and Osteopontin, as requested. As the original manuscript included qRT-PCR data on Bone sialo-protein II (Ibsp), the corresponding protein expression data has also been added (Figure 5) as well.

We are including a new change to the financial disclosure statement from Orthofix- this has been included in the revised manuscript.

**Finance Disclosure and Conflicts of Interest:**

This study was supported by a research grant received by JAD from Orthofix Medical Inc. (Lewisville TX, USA). JAD and NSK received salary support from this grant. Data acquisition, analysis, and interpretation were done by JAD, NSK and DF solely based on their scientific discretion. Well-designed and documented laboratory protocols were strictly followed to ensure scientific soundness of the study. The publication of these research findings was a result of the Principal Investigator’s academic judgement. JTR, EIW and NZ are employees of and own stocks in Orthofix Medical Inc.

We feel that now, with all requests by the reviewer’s having been satisfied, the manuscript is now ready and deserving of publication in PLOS ONE.

Best Regards,

Joseph DiDonato, PhD
